# Elian granules alleviate precancerous lesions of gastric cancer in rats by suppressing M2-type polarization of tumor-associated macrophages through NF-κB signaling pathway

**DOI:** 10.1186/s12906-023-04015-7

**Published:** 2023-06-08

**Authors:** Zhirong Yi, Qingling Jia, Yujiao Wang, Yuqin Zhang, Tianyi Xie, Jianghong Ling

**Affiliations:** 1 Department of Traditional Chinese Medicine, The First Affiliated Hospital of Guangxi Medical University, Guangxi Medical University, Nanning, 530021 China; 2 Department of Gastroenterology, Shuguang Hospital, Shanghai University of Traditional Chinese Medicine, Shanghai, 200021 China

**Keywords:** Elian granules, Precancerous lesions of gastric cancer, M2-type tumor-associated macrophages, Polarization, NF-κB signaling pathway

## Abstract

**Background:**

Precancerous lesions of gastric cancer (PLGC) refer to a kind of histopathological changes in the gastric mucosa that can progress to gastric cancer. Elian granules (ELG), a Chinese medicinal prescription, have achieved satisfactory results in the treatment of PLGC. However, the exact mechanism underlying the therapeutic effect of ELG remains unclear. Here, this study aims to explore the mechanism of ELG alleviating PLGC in rats.

**Methods:**

The chemical ingredients of ELG were analyzed using ultra-performance liquid chromatography-tandem mass spectrometry (UPLC-MS). Specific Pathogen Free SD rats were randomly assigned to 3 groups: the control, model, and ELG groups. The 1-Methyl-3-nitro-1-nitrosoguanidine (MNNG) integrated modeling method was adopted to construct the PLGC rat model in groups except for the control group. Meanwhile, normal saline was used as an intervention for the control and model groups, and ELG aqueous solution for the ELG group, lasting 40 weeks. Subsequently, the stomach of rats was harvested for further analysis. Hematoxylin-eosin staining of the gastric tissue was conducted to assess the pathological changes. Immunofluorescence was carried out for the expression of CD68, and CD206 proteins. Real-time quantitative PCR combined with Western blot was conducted to analyze the expression of arginase-1(Arg-1), inducible nitric oxide synthase (iNOS), p65, p-p65, nuclear factor inhibitor protein-α (IκBα), and p-IκBα in gastric antrum tissue.

**Results:**

Five chemical ingredients including Curcumol, Curzerenone, Berberine, Ferulic Acid, and 2-Hydroxy-3-Methylanthraquine were identified in ELG. The gastric mucosal glands of rats treated with ELG were orderly arranged, with no intestinal metaplasia and no dysplasia. Furthermore, ELG decreased the percentage of M2-type TAMs marked with CD68 and CD206 proteins, and the ratio of Arg-1 to iNOS in the gastric antrum tissue of rats with PLGC. In addition, ELG could also down-regulate the protein and mRNA expression of p-p65, p65, and p-IκBα, but up-regulate the expression of IκBα mRNA in rats with PLGC.

**Conclusions:**

The results showed that ELG attenuates PLGC in rats by suppressing the M2-type polarization of tumor-associated macrophages (TAMs) through NF-κB signaling pathway.

## Background

Gastric cancer is an important risk factor threatening human health. According to the global cancer data statistics, gastric cancer ranks fifth among all cancer incidence rates and accounts for the third leading cause of cancer-related deaths [1]. Correa believes that gastric cancer develops gradually from normal gastric tissue through multiple stages of pathological evolution, including chronic gastritis, chronic atrophic gastritis, intestinal metaplasia, and dysplasia, among which the last three stages belong to precancerous lesions of gastric cancer (PLGC) [2]. However, the exact molecular mechanism of the pathological evolution has not been clarified. Previous studies have shown that tumor-associated macrophages (TAMs) in the tumor microenvironment exert an important influence on the formation and development of gastric cancer. M2-type TAMs can inhibit anti-gastric cancer immune response, promote gastric cancer angiogenesis, and accelerate cancer cell proliferation, infiltration, and metastasis, which is positively correlated with poor prognosis [3–5]. However, whether they are involved in the mechanism of the formation of PLGC needs further confirmation. Moreover, it has been found that continuously abnormal upregulation of the nuclear factor-kappa B (NF-κB) pathway is common in PLGC [6], which inhibits apoptosis of atypical cells and promotes cell proliferation by regulating the transcription of such downstream proliferation and apoptosis-related target genes as Bcl-2, Bax, and c-Myc, thus leading to the formation of PLGC [7, 8].

Traditional Chinese medicine characterized by complex and diverse ingredients has an obvious effect on the reversal of pathological changes in PLGC by multi-targets and multi-signaling pathways [9, 10]. Elian granules (ELG), also known as Leweijian, is a Chinese medicinal formulation composed of 12 Chinese herb medicines (Table 1) and has achieved good results in the treatment of PLGC by alleviating chronic inflammation, atrophy, and intestinal metaplasia of gastric mucosa [11, 12]. Based on the network pharmacology and animal experiments, we had verified that ELG could effectively act on PLGC rats and reverse its pathological changes [13]. In addition, previous studies had also found that ELG could promote the apoptosis of atypical cells in gastric tissue of PLGC rats via down-regulating the anti-apoptosis protein Bcl-2 and upregulating the Fas protein, and improve the pathological changes of gastric cancer rats to a certain extent [14, 15]. However, it had not been discussed whether the action mechanism of ELG was related to the M2-type polarization of TAMs and the involvement of the NF-κB pathway.

**Table 1:** Herbs in ELG

Latin name	Chinese name	Medicinal part	Dosage (g)
*Curcuma phaeocaulis* VaL.	Ezhu	Rhizoma	15
*Coptis chinensis* Franch.	Huanglian	Rhizoma	3
*Salvia miltiorrhiza* Bge.	Danshen	Radix et Rhizoma	10
*Hedyotis diffusa* Willd.	Baihuasheshecao	All	30
*Angelica sinensis* (Oliv.) Diels	Danggui	Radix	10
*Codonopsis pilosula* (Franch.) Nannf.	Dangshen	Radix	10
*Glycyrrhiza uralensis* Fisch.	Gancao	Radix et Rhizoma	6
*Atractylodes macrocephala* Koidz.	Baizhu	Rhizoma	10
*Pinellia ternate* (Thunb.) Breit.	Banxia	Rhizoma	10
*Poria cocos* (Schw.) Wolf	Fuling	Sclerotia	12
*Taraxacum mongolicum* Hand.-Mazz.	Pugongying	All	30
*Citrus reticulata* Blanco	Chenpi	Peel	6

**Fig. 1:**
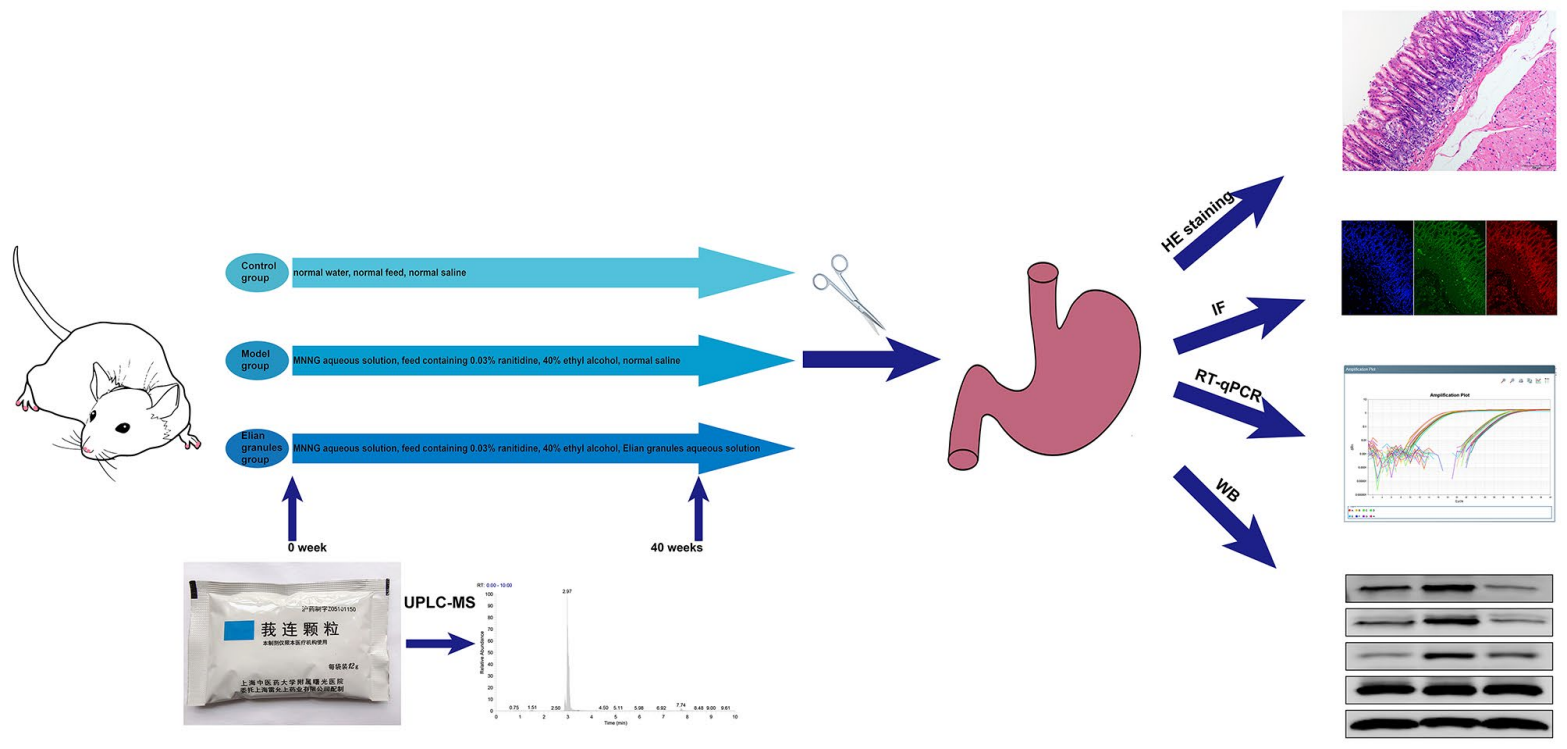
The flow chart of this study design. HE staining: Hematoxylin-eosin staining; IF: Immunofluorescence; MNNG: 1-Methyl-3-nitro-1-nitrosoguanidine; RT-qPCR: real-time quantitative polymerase chain reaction; UPLC-MS: ultra-performance liquid chromatography-tandem mass spectrometry; WB: Western blot

In this study, ELG of which the chemical ingredients were identified by ultra-performance liquid chromatography-tandem mass spectrometry (UPLC-MS) was given to the PLGC rat constructed using the 1-Methyl-3-nitro-1-nitrosoguanidine (MNNG) integrated modeling method. Hematoxylin-eosin (HE) staining of the gastric tissue was conducted to assess the pathological changes. Immunofluorescence, real-time quantitative PCR (RT-qPCR), and Western blot (WB) experiments were then performed to investigate the effects of ELG on the M2-type polarization of TAMs and the NF-κB pathway in PLGC rats (Fig. 1). We hope this will elucidate the mechanism of occurrence and development of PLGC and provide novel therapeutic strategies.

## Materials and methods

### Preparation of drug aqueous solution

ELG (Lot. number: Z05101150) was obtained from Shuguang Hospital affiliated to Shanghai University of Traditional Chinese Medicine and identified by Li Liu, the Director of Pharmacy of Shuguang Hospital. ELG was fully dissolved in deionized water to 0.324 g/mL, then heated at a constant temperature of 100℃ for 10 min, and cooled to room temperature for intragastric administration. The selection of ELG dose was based on the comparison of efficacy between multiple doses and converted from human dose to rat dose [16].

### UPLC-MS analysis of ELG

100 mg of ELG sample accurately weighed by an electronic balance were ground for 5 min with 1000 µL methanol, followed by vortex mixing for 10 min and centrifugation at 4℃ for 10 min with 20,000 ×g centrifugal force. The supernatant was filtered for further analysis. Subsequently, UPLC was conducted on an UltiMate 3000 RS liquid chromatography (Thermo Scientific, USA). A (0.1% formic acid) and B (0.1% acetonitrile) were used together as the mobile phase. Chromatographic separation of ELG sample solution was carried out using AQ-C18 chromatographic column (150 × 2.1 mm, 1.8 μm). Table 2 showed the gradient elution procedures. The flow rate was 0.3ml/min during separation with the column temperature and automatic sampler temperature set at 35℃ and 10℃, respectively. The injection volume was 5ul. Then, the mass spectrometry experiments were performed to further identify the constituents of Elian granules on a mass spectrometer (Q Exactivemass, Thermo Scientific, USA) which ionized the sample using the electrospray ionization source, and the parallel reaction monitoring mode is used for ion detection. The operating parameter was set as follows: 3.8 kV for spray voltage, 300 and 350 °C for the capillary and auxiliary gas heater temperature, respectively, 40 and 10 (arbitrary units) for the flow rates of the sheath and auxiliary gas, respectively. Finally, mass spectrometer data were analyzed using Xcalibur 4.1 software.

**Table 2:** Gradient elution procedures

Time (min)	0.1% formic acid (V%)	0.1%acetonitrile (V%)
0.0	95	5
2.0	35	65
6.0	5	95
8.0	5	95
8.5	95	5
10.0	95	5

### Animal experiments

6-week-old SPF male SD rats were produced by the Experimental Animal Center of Guangxi Medical University, weighing 180–220 g, and raised in a suitable temperature, humidity, and light condition. All the operation of animals meets the requirements of the Animal Ethics Review Committee of Guangxi Medical University (NO. 202,007,054). 18 SPF male SD rats were randomly assigned to the control, model, and ELG groups. Except for the normal group fed with normal feed and water, the model and ELG groups adopted MNNG (CAS NO.: 70-25-7, Aladdin Biochemical Technology Co., Ltd., Shanghai, China) integrated modeling method to construct the PLGC rat model [17, 18]: (1) MNNG solution (200 µg/mL) protected from light could be drunk freely; (2) Rats were fed with 0.03% ranitidine feed (Keao Xieli feed Co., Ltd., Beijing, China) for 48 h and fasted for 24 h; (3) 40% ethanol (10 ml/kg/d) was administered to hurt the gastric mucosa by gavage on the afternoon of the fast. Meanwhile, the control group and model group were treated with normal saline (10 ml/kg/d), while the ELG group interfered with ELG aqueous solution (3.240 g/kg/d) by intragastric administration. The intervention lasted for 40 weeks. After the last administration and feed, the three groups of rats fasted for 24 h. The entire stomach of all the rats anesthetized with 1.5% pentobarbital sodium (3 ml/kg) was dissected, cut along the greater curvature, and then cleaned with low-temperature deionized water. Tissue strips of 20 mm × 3 mm from the lesser curvature of the gastric antrum of all the rats were fixed with 4% paraformaldehyde solution and used to make paraffin sections. The remaining gastric tissue was frozen in two parts at -80℃ for subsequent detection.

### HE staining

Paraffin Sect. (4 μm) made by paraffin slicer (RM2235, Leica, Germany) were dewaxed and hydrated by xylene, graded ethanol, and pure water. After staining with hematoxylin and eosin, the paraffin sections were dehydrated by graded ethanol, vitrified with xylene, and sealed with neutral resin. Then, the pathological changes in gastric tissue were assessed using the optical microscope (200 ×).

### Immunofluorescence

Dewaxed and hydrated paraffin sections were immersed in ethylene diamine tetraacetic acid and the antigen repair was carried out in a microwave heating mode. After the endogenous peroxidase was quenched using 3% hydrogen peroxide, the samples were blocked with 5% BSA for 30 min and then incubated with CD206 antibody (1:1,000, Cat NO.: sc-70,586, Santa Cruz Biotechnology, CA, USA) in a wet box at 4 °C overnight. Subsequently, after being washed with PBS, the sections were incubated with the HRP conjugated secondary antibody (1:500, Cat NO.: GB23301, Servicebio technology Co., Ltd., Wuhan, China) at room temperature for 50 min, followed by incubation with Cy3-Tyramide (1:2,000, Cat NO.: G1223, Servicebio technology Co., Ltd., Wuhan, China) under dark conditions for 10 min. After being stripped of primary and secondary antibodies, all the sections were incubated with CD68 antibody (1:100, Cat NO.: sc-70,761, Santa Cruz Biotechnology, CA, USA) and then combined with the Alexa Fluor® 488-conjugated secondary antibody (1:400, Cat NO.: GB25301) produced by the same manufacturer as Cy3-Tyramide just as the above process. Finally, the nucleus was displayed using 4′,6-diamidino-2-phenylindole at room temperature under dark conditions for 5 min before the slides were sealed with the anti-fluorescence quenching agent for further observation using the forward fluorescence phase-contrast microscopy imaging system (BX53F, Olympus, Japan).

### RT-qPCR test

Total RNA in the gastric antrum tissue was extracted and reversely transcribed into cDNA (reaction conditions: Step 1: 37℃, 15 min; Step 2: 85℃, 5 s; Step 3: 4℃). 10 ul total reaction system consisting of cDNA template, TB Green II (Cat NO.: RR820A, Takara Bio Inc., Japan), Rox, primers (Sangon Biotech, Shanghai, China) (Table 3), and RNase-free water were prepared for RT-qPCR amplification (amplification conditions: stage 1: 95℃, 30 s, 1 cycle; stage 2: 95℃, 5 s, 60℃, 34 s, 40 cycles). 2^−ΔΔCt^ represented relative mRNA expression and was used for statistical analysis.

**Table 3:** Primers for RT-qPCR test

Gene	sequence	length (bp)
Arg-1	Forward: cag tat tca ccc cgg cta	150
	Reverse: cct ctg gtg tct tcc caa	
iNOS	Forward: cgg aga aca gca gag ttg g	149
	Reverse: gga ata gca cct ggg gtt t	
NF-κB(p65)	Forward: ttc ctg ggg aga gaa gca	114
	Reverse: ggt gag gtg ggt ctt tgg	
IκBα	Forward: cat ctc cac tcc gtc ctg	110
	Reverse: gca ccc aaa gtc acc aag	
β-Actin	Forward: cct cac tgt cca. cct tcc a	120
	Reverse: ggg tgt aaa acg cag ctc a	

### WB analysis

The protein of the gastric antrum tissue in each group was extracted for further detection of the concentration using the protein assay kit, then separated using 10% SDS-polyacrylamide gels for subsequent transfer to the PVDF membranes activated by methyl alcohol. After being blocked with 5% BSA at room temperature for 1 h, the membranes containing the protein to be tested were incubated with corresponding primary antibodies prepared using an antibody diluent, including p-p65 (1:1,000, Cat NO.: 3033), p65 (1:1,000, Cat NO.: 8242), IκBα (1:500, Cat NO.: 4814), Arg-1 (1:500, Cat NO.: 93,668), β-actin (1:1,000, Cat NO.: 4970) produced by Cell Signaling Technology (MA, United States), p-IκBα (1:500, Cat NO.: BSM-52169R) manufactured by Bioss Biotechnology Co., Ltd. (Beijing, China), iNOS (1:500, Cat NO.: ab15323) from Abcam (United Kingdom), at 4℃ overnight. After being washed with TBST for 10 min × 3 times, all the membranes were incubated with the corresponding fluorescein second antibody (1:10,000, Cat NO.: 5151, Cell Signaling Technology, MA, USA) for 1 h. Finally, the protein blots were detected using the infrared fluorescence imaging system (Odyssey CLx, LI-COR, USA). The gray values of each protein band detected by Image J software were normalized with that of the internal reference protein for statistical analysis.

### Statistical analysis

The measurement data were analyzed by the SPSS20.0 software and expressed as mean ± SD. Statistical differences in data between multiple groups were determined by one-way ANOVA. P < 0.05 was taken as the criterion to judge whether the difference was statistically significant. Finally, the statistical diagram was conducted using GraphPad Prism 8.

## Results

### Effective chemical ingredients of ELG

**Fig. 2:**
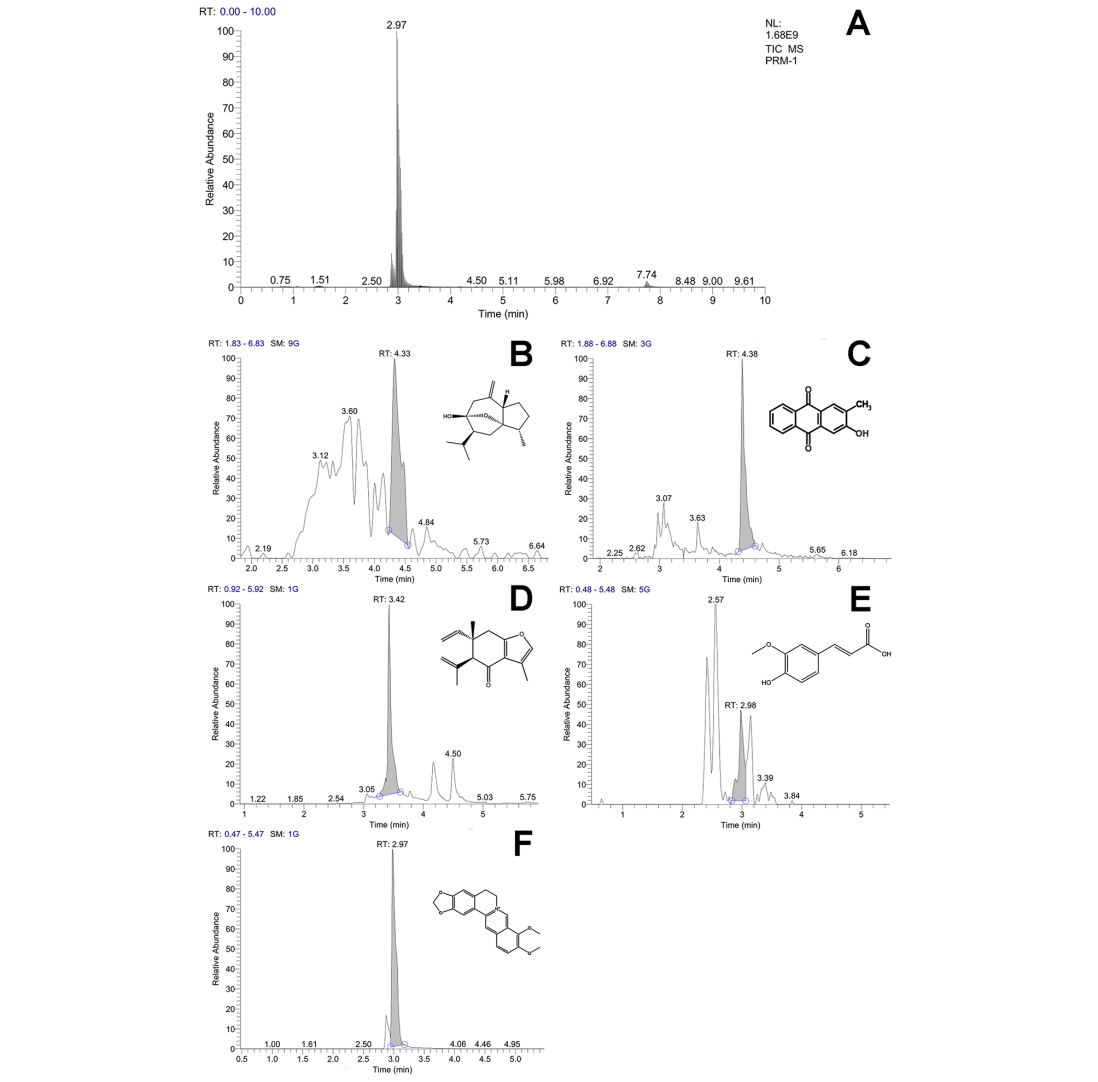
Five chemical ingredients in ELG were identified by UPLC-MS. (**A**) Total ion current diagram of ELG. (**B**) curcumol. (**C**) 2-hydroxy-3-methylanthraquinone. (**D**) Curzerenone. (**E**) Ferulic acid. (**F**) Berberine

UPLC-MS was used to analyze the chemical ingredients of ELG. Five effective ingredients including Curcumol, Curzerenone, Berberine, Ferulic Acid, and 2-Hydroxy-3-Methylanthraquine were identified, which were derived from Curcumae Rhizoma, Coptidis Rhizoma, Angelicae Sinensis Radix, and Hedyotis Diffusa, respectively (Table 4). The total ion current diagram and the respective retention times were shown in Fig. 2.

**Table 4:** Five chemical ingredients identified in ELG

Ingredients	Molecular formula	Molecular mass	Charged property	MS1	Retention time (min)	Herbs
Curcumol	C_15_H_24_O_2_	236.177	+	237.185	4.33	*Curcuma phaeocaulis* VaL.
Curzerenone	C_15_H_18_O_2_	230.130	+	231.138	3.42	*Curcuma phaeocaulis* VaL.
Berberine	C_20_H_18_NO_4_	336.123	+	336.123	2.97	*Coptis chinensis* Franch.
Ferulic Acid	C_10_H_10_O_4_	194.057	+	195.065	2.98	*Angelica sinensis* (Oliv.) Diels
2-Hydroxy-3-Methylanthraquine	C_15_H_10_O_3_	238.062	+	239.070	4.38	*Hedyotis diffusa* Willd.

### ELG improved gastric histopathological changes in PLGC rats

**Fig. 3:**
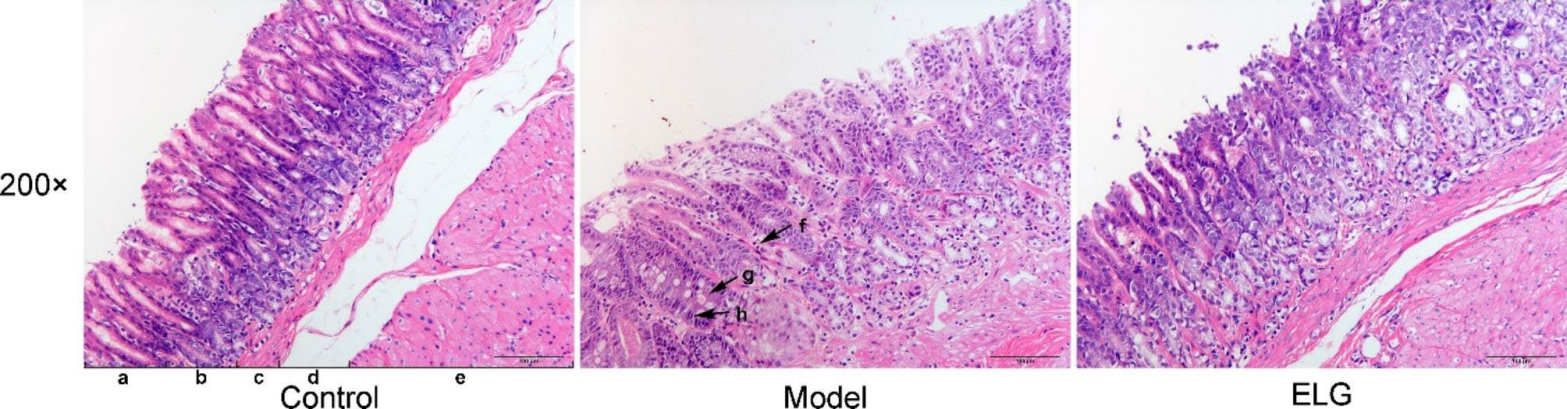
ELG alleviated pathological changes in the gastric tissue of rats with PLGC. a: epithelium; b: lamina propria; c: muscularis mucosa; d: submucosa; e: muscularis propria; f: inflammatory cell; g: intestinal metaplasia; h: dysplasia. (Hematoxylin-eosin staining, 200 ×, Scale bar = 100 μm) (*n* = 6)

Then, the effect of ELG on the pathological changes of gastric tissue in PLGC rats was assessed using HE staining. The results show that the gastric mucosa glands of the rats in the control group were arranged regularly with unified morphology. In addition, no atrophic gland, no intestinal metaplasia, no dysplasia cells, and no inflammatory cells were observed in this group. Interestingly, the structure of the gastric mucosa glands was disordered, atrophic glands, intestinal metaplasia, and dysplasia were observed, and inflammatory cells infiltrated gastric tissue to varying degrees in the model group, suggesting that the construction of the rat model of PLGC was successful. The gastric mucosa glands of rats treated with ELG were orderly arranged with normal morphology and no atrophic gland, no intestinal metaplasia, no dysplasia, and no obvious inflammatory reaction were observed (Fig. 3).

### ELG suppressed M2-type polarization of TAMs in PLGC rats

**Fig. 4:**
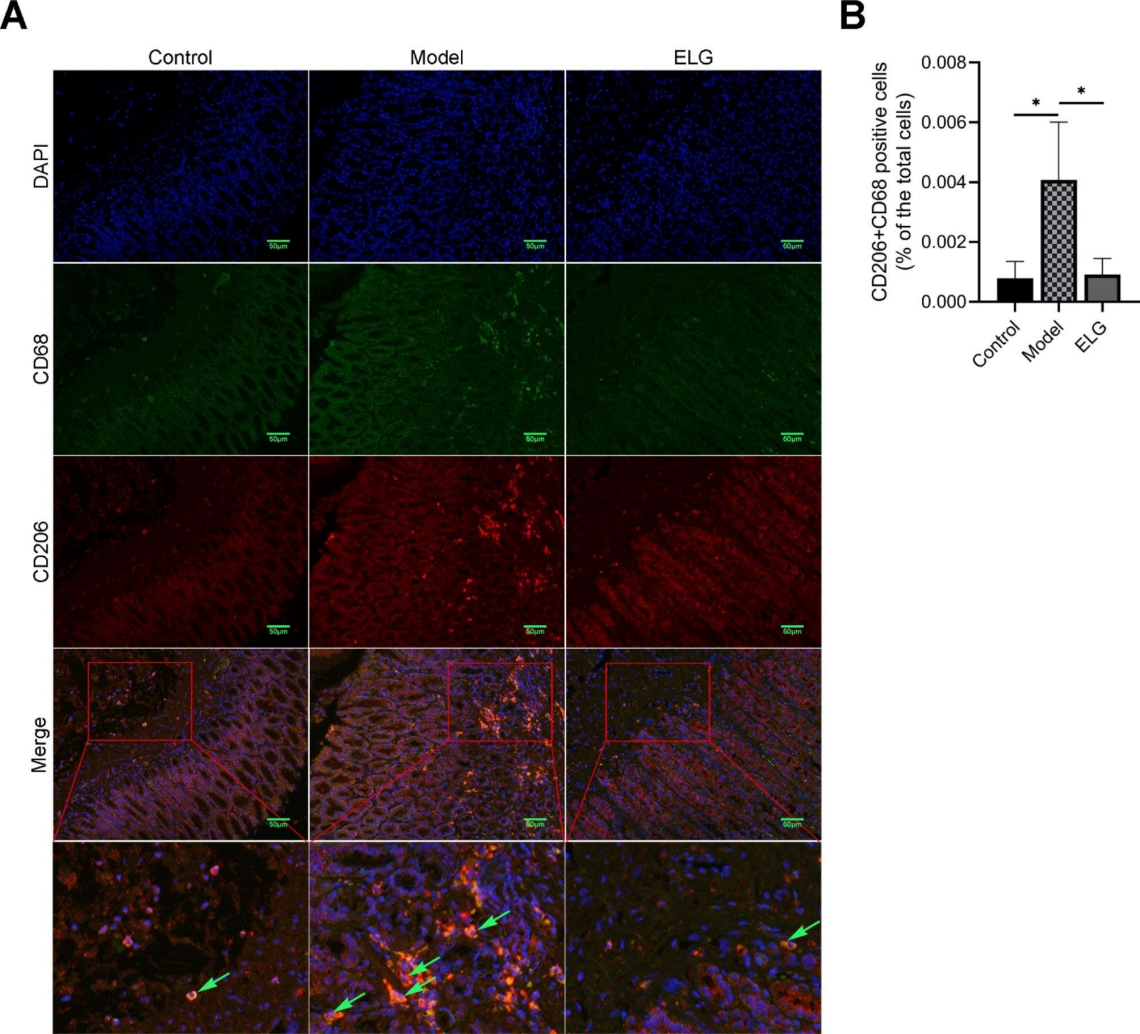
ELG suppressed M2-type macrophages in gastric antrum tissue of rats with PLGC. (**A**) Immunofluorescence detection of CD68 and CD206 proteins of all groups (200 ×) (*n* = 6). (**B**) Percentage of macrophages expressing CD68 and CD206 proteins in total cells in each group (*n* = 6). Data were represented by the mean and standard deviation, one-way ANOVA. Differences with *P* < 0.05 were considered statistically significant. ^*^P < 0.05

**Fig. 5:**
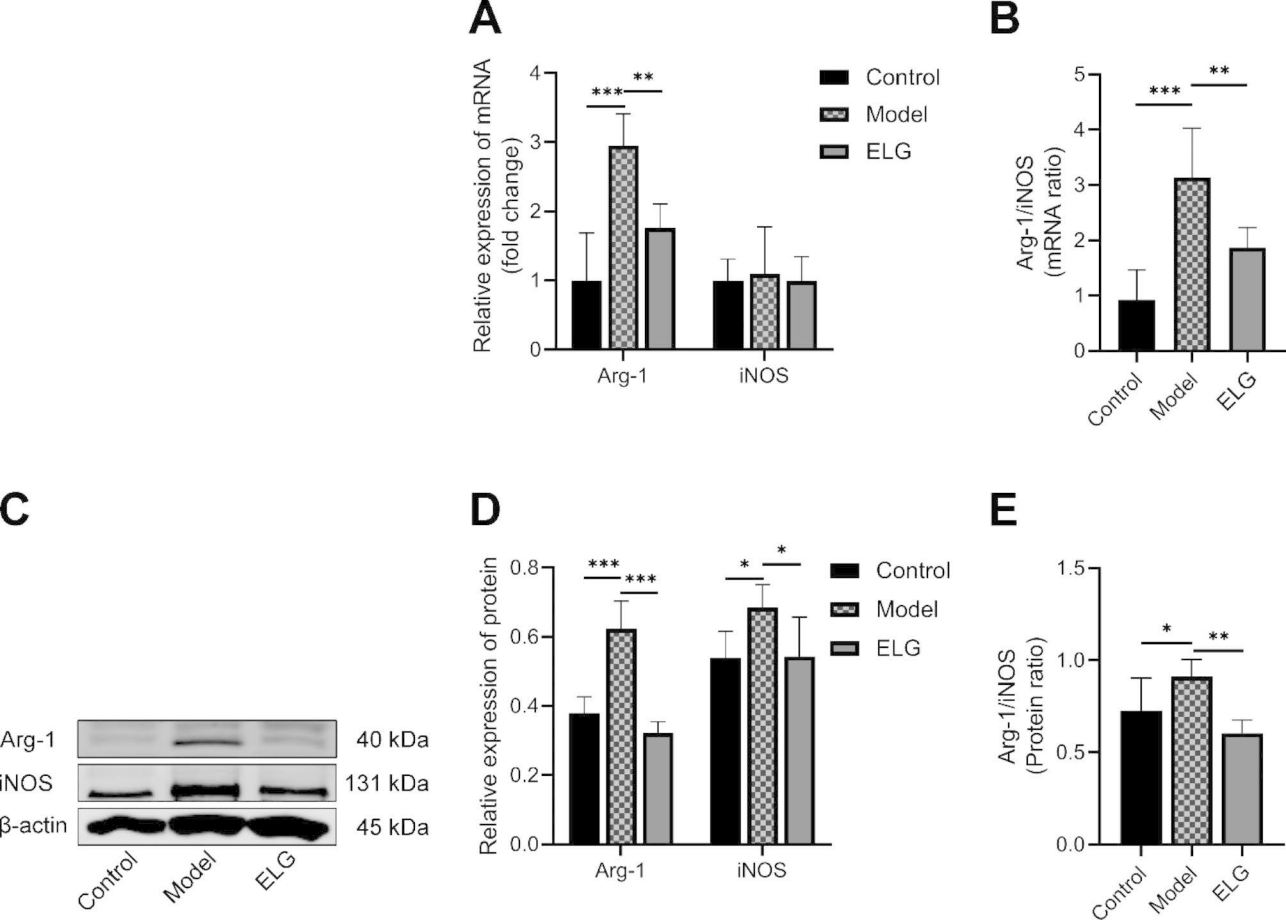
ELG inhibited M2-type polarization of macrophages in gastric antrum tissue of rats with PLGC. (**A**) The Arg-1 and iNOS mRNA expression of all groups (*n* = 6). (**B**) The ratio of Arg-1 mRNA to iNOS mRNA representing the M2-type polarization of macrophages (*n* = 6). (**C-D**) The Arg-1 and iNOS protein expression of all groups was detected by WB (*n* = 6). (**E**) The ratio of Arg-1 protein to iNOS protein representing the M2-type polarization of macrophages (*n* = 6). Data were represented by the mean and standard deviation, one-way ANOVA. Differences with *P* < 0.05 were considered statistically significant. ^*^P < 0.05, ^**^P < 0.01, ^***^P < 0.001

To investigate the inhibitory effects of ELG on M2-type polarization of TAMs, immunofluorescence, RT-qPCR, and WB detections of the macrophage polarization-related indexes in rat gastric antrum tissue, such as CD68, CD206, Arg-1 (M2-type TAMs markers), iNOS (M1-type TAMs marker), and the ratio of Arg-1 to iNOS were conducted. As the immunofluorescence results showed, compared with the model group, the percentage of M2-type TAMs of PLGC rats treated with ELG was significantly decreased (Fig. 4A,B). Meanwhile, the results of WB and RT-qPCR showed that ELG could down-regulate the expression of Arg-1 and iNOS (Fig. 5A,C,D). More importantly, ELG could reduce the ratio of Arg-1 to iNOS, an index of M2-type polarization of TAMs (Fig. 5B,E).

### ELG inhibited the activity of the NF-κB signaling pathway in PLGC rats

**Fig. 6:**
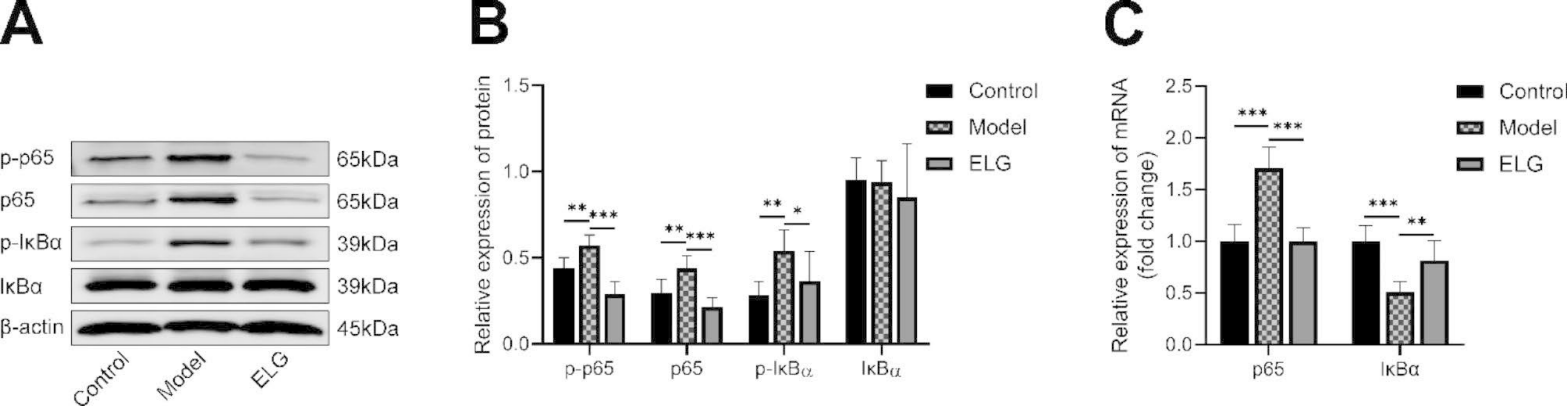
ELG inhibited the NF-κB signaling pathway of rats with PLGC. (**A-B**) The p-p65, p65, p-IκBα, and IκBα protein expression of all groups was detected by WB (*n* = 6). (**C**) The p65 and IκBα mRNA expression of all groups (*n* = 6). Data were represented by the mean and standard deviation, one-way ANOVA. Differences with *P* < 0.05 were considered statistically significant. ^*^P < 0.05, ^**^P < 0.01, ^***^P < 0.001

To further explore the underlying signaling pathway by which ELG alleviated PLGC in rats, the indicators of the NF-κB pathway, including p-p65, p65, p-IκBα, and IκBα, were detected using RT-qPCR and WB. Finally, we found that the expression of p65, p-p65, and p-IκBα were all down-regulated in PLGC rats after treatment with ELG, while the expression of IκBα mRNA was up-regulated (Fig. 6A-C).

## Discussion

Traditional Chinese medicine consisting of multi-components could play a beneficial role in reversing the pathological changes of PLGC via multi-targets and multiple signaling pathways with few side effects, thus having certain advantages in the treatment of PLGC [19–21]. ELG was modified from Chenxia-Liujunzi Decoction by Professor CAI Gan, a famous traditional Chinese medicine expert, and had been verified by the clinical application that it could significantly attenuate PLGC [12]. Moreover, studies on the pharmacology of Traditional Chinese medicine have found that the components of ELG can inhibit the proliferation of heteromorphic cells, such as curcumol, an ingredient of Curcuma phaeocaulis VaL., could induce mitochondrial apoptosis and then promote apoptosis of cancer cells [22]. Curzerenone, another component of Curcuma phaeocaulis VaL., also plays an anticancer role in drug-resistant lung cancer cells through programmed cell death [23]. Ferulic acid, one of Angelica sinensis (Oliv.) Diels’s main components, could block the cell cycle of cervical cancer cells [24]. Hedyotis diffusa Willd. can induce apoptosis and inhibit the proliferation of He-La cells of cervical cancer by inhibiting telomerase activity and Ki-67 expression [25]. 2-hydroxy-3-methylanthraquinone in the Hedyotis diffusa Willd. inhibits lung cancer cells by regulating the IL-6-induced JAK2/STAT3 pathway [26]. Berberine, the active ingredient of Coptis chinensis Franch., can exert an anti-tumor effect on human ovarian cancer cells by stimulating the pro-apoptotic mechanism [27]. Interestingly, in this study, HE staining results revealed that ELG could alleviate the intestinal metaplasia and dysplasia of gastric mucosa in rats with PLGC, which was in line with the above pharmacological results of Traditional Chinese medicine.

Subsequently, we investigate whether M2-type TAMs were associated with the formation of PLGC and the mechanism of the therapeutic effect of ELG. Previous studies have found that the invasion of TAMs in the tumor microenvironment is involved in the occurrence of gastric cancer. TAMs are mainly divided into two types: classical activated TAMs (M1-type TAMs) and alternative activated TAMs (M2-type TAMs). M1-type TAMs expressing CD68 and CD86 markers, secreting pro-inflammatory cytokines and chemokines, and uniquely expressing iNOS could promote an inflammatory response. M2-type TAMs expressing CD68 and CD206 markers and Arg-1 could promote the formation, proliferation, and metastasis of tumors through multiple pathways [28, 29]. M2-type TAMs can mediate immune escape of heterotypic cells through immunosuppression, resulting in the continued survival of heterocyst [30]. Furthermore, M2-type TAMs can also regulate the VEGF and VEGF-C expression in the gastrointestinal tumor microenvironment through NF-κB, promote angiogenesis, and provide energy and oxygen for the heterocyst [31]. In the previous study on the molecular mechanism of the action of Elian granules on precancerous lesions of gastric cancer through network pharmacology, we found that the active components of Elian granules can regulate the activation factors of M2-type macrophages, such as IL-4 and IL-10 [13]. For instance, the effective components of *Coptis chinensis Franch* can act on the IL-10 target which then activates M2-type macrophages. Berberine in *Coptis chinensis Franch* can also directly affect the expression level of iNOS in macrophages, thus regulating the physiological function of macrophages. In this study, the immunofluorescence results indicated that M2-type TAMs were indeed involved in PLGC and ELG could alleviate PLGC by restraining the infiltration of M2-type TAMs. Moreover, it was found from the WB and RT-qPCR results that ELG could reduce Arg-1, iNOS, and the ratio of Arg-1 to iNOS, an indicator of M2-type polarization of TAMs, which suggested that ELG exerted a beneficial effect on PLGC by blocking the invasion of TAMs, mainly by inhibiting the M2-type polarization of TAMs.

The tumor-promoting effect of M2-type TAMs is precisely regulated by intracellular and extracellular signals, in which nuclear transcription factor NF-κB affects the biological function of M2-type TAMs in many ways. NF-κB family members (RelA, RelB, cRel, p50, and p52) often interact with IκB and exist in the cytoplasm in an inactivated state. In classical pathways, IKK activation by various pathways could result in phosphorylation and degradation of IκBα and release of the NF-κB dimer which might combine with the promoter sequences of specific target genes in the nucleus to exert a transcriptional activation effect [32]. NF-κB signaling pathway regulates the transcriptional activity of many downstream target genes and is related to multiple biological functions such as inflammation, immunosuppression, angiogenesis, and tumor proliferation [33]. Studies have shown that NF-κB can recruit TAMs to the tumor microenvironment through chemokine (C-C motif) ligand 2 (CCL2) [34], and regulate the M2-type polarization of TAMs [35], promoting tumor progression. At the same time, the tumor-promoting effect of M2-type TAMs also depends on the NF-κB pathway [36]. NF-κB mediates the immunosuppressive effect of M2-type TAMs, causing atypical cells to escape immune surveillance and facilitating the formation of tumors [37]. NF-κB is involved in M2-type TAMs-induced tumor angiogenesis and provides energy for tumor cells [38]. Furthermore, it has been confirmed that the NF-κB pathway may play a crucial part in the transformation of PLGC into gastric cancer directly [39]. Continuous up-regulation of the NF-κB signaling pathway can affect apoptosis-related genes and promote the transcription of cell cycle-related protein, resulting in the disorder of dynamic balance between cell proliferation and apoptosis and thus promoting the progression of PLGC [7, 40]. Interestingly, down-regulation of the NF-κB pathway helps to inhibit the progression of PLGC [6, 41, 42]. To support this viewpoint, WB and RT-qPCR were used in this study to analyze the expression of p65, p-p65, IκBα, and p-IκBα, the indicators of the NF-κB pathway. Finally, we found the NF-κB pathway was indeed responsible for the PLGC in rats, and ELG could block the occurrence and development of PLGC by inhibiting the NF-κB pathway, which is consistent with the results of previous network pharmacological study and may be related to the upstream MAPK signaling pathway and PI3K signaling pathway [13].

## Conclusions

To sum up, ELG can improve the gastric histopathological changes in rats with PLGC, and the underlying mechanism is related to the suppression of M2-type polarization of TAMs through NF-κB signaling pathway, thus shedding light on a new direction for the treatment of PLGC. Since the mechanism of action of ELG is complex for the abundant action targets and pathways, we may be able to further clarify the underlying mechanism of its effect on PLGC through molecular docking, network pharmacology analysis, gene knockout verification, and other technical methods in future studies.

## Data Availability

The data used in the present study are available from the corresponding author upon reasonable request.

## Abbreviations

Arg-1: Arginase-1
ELG: Elian granules
HE: Hematoxylin-eosin
IκBα: Inhibitor kappa B alpha
iNOS: Inducible nitric oxide synthase
MNNG: 1-Methyl-3-nitro-1-nitrosoguanidine
NF-κB: Nuclear factor-kappa B
PLGC: Precancerous lesions of gastric cancer
RT-qPCR: Real-time quantitative PCR
SPF: Specific pathogen-free
TAMs: Tumor-associated macrophages
UPLC-MS: Ultra-performance liquid chromatography-tandem mass spectrometry
WB: Western blot
